# Medicine, Pathological, Practical, and Therapeutical

**Published:** 1836-10

**Authors:** 


### MEDICINE,

### PATHOLOGICAL, PRACTICAL, AND THERAPEUTICAL.

### *On Pneumonia of the Old*, by MM. HOURMANN and DECHAMBRE.

[This is a continuation of the researches made at the Salpetrière on the diseases of the aged. The previous memoirs on the natural changes of structure and functions of the lungs, on the pulse, &c. we have analysed in No. I. p. 233, and No. II. p. 553.]

1. *Anatomical characters.* Pneumonia is the most frequent and dangerous of the acute diseases to which the old are subject. Its diagnosis, so evident in the adult, is difficult, as the affection is often overlooked from the apparent mildness of the general symptoms; or obscured from the utter prostration caused by the attendant adynamic fever. The changes it produces are peculiar. Independently of inflammation, the vessels of the lungs of the old are always more or less congested, so that it is always difficult to establish the existence of pneumonia, in its commencement or even progress, in the dead body, if the symptoms during life have not been carefully observed.

I. *Simple congestion.* There are three degrees: in the first, the lungs, which are intensely red, both crepitate and swim completely; they contain a great quantity of bloody, frothy serum; there is no change of consistence: in the second degree, their colour is deeper: in the third, they are livid, the lobules are nearly confounded; there is an increase of volume; their cohesion is diminished; a blackish fluid with bubbles of air escapes from an incision; they slightly crepitate, and float less high in water. Dried sections of congested lungs show that the cells are still permeable, but contracted in proportion to the congestion; this is much less marked when the cells are torn and irregular. *Cæteris paribus*, the arborizations on the sides of the cells diminish in proportion to the rarefaction of the tissue, as might be expected from the previous observations on structure, (No. 1.)

II. *Congestion with imperviousness of the pulmonary parenchyma.* There are two varieties; one in which the granular appearance, regarded as peculiar to pneumonia, is absent, and another in which it is well marked:—1*st form.* The pulmonary tissue is of a dark colour, often blue or black, and a section is homogeneous, and remarkably polished; sometimes it is elastic like caoutchouc; at others easily broken up. On cutting the lung, a fluid or viscid liquid escapes, often reddish, but not frothy. Compression increases the suppleness and elasticity of the first portions, and, if after compression they are dried, the cells reappear, without having experienced any other change than contraction, which is seen by a lens to be produced by congestion of the vessels running in the intervals, separating the cells, thickening the membranes dividing them, and less evident in the cells themselves. 2*d form.* (*Red hepatization.*) The granulations are either regular, well marked, and much larger than in the pneumonia of adults, or less defined, running into one another. Generally there is less friability than in adults; the lung being resistant and easily cut into thin and flexible slices; it is not so heavy, nor does it sink so deeply. This lightness may be owing to the rarefaction which the lungs undergo with age.

III. *Suppuration of the Lung.* There are two varieties; one without granulations, and the other granulated. In the first form there are two varieties: (1,) lines or patches of a greyish white colour are seen in the middle of the congested lung, which appear to be pus beneath a fine membrane: on making pressure with the nail, the pus can be deplaced, and made to pass into the pulmonary tissue as far as the surface where it appears to transude; the texture then regains its suppleness, and on drying it the cells reappear. (2). The texture appears like granite, being a singular mixture of red and dull white; the pus being deposited in spots of one or two lines in diameter. Pressure with the nail does not displace it, but it is removed easily on the point of a lancet, and is of the consistence of coagulated albumen, and never fluid. When thus extracted, it is evident that it is contained in irregular cells whose walls are of a deep red colour. It is never prolonged into the small bronchial ramifications.

*Suppuration with granulations (Grey hepatization,)* is much more frequent. The granulations are larger than in the adult, though less than in the red stage. The granulations may disappear and be replaced by small abscesses, but extensive abscesses of the lungs are excessively rare. The lung is extremely friable, the slightest pressure reducing it to a pulp, from which pus flows in abundance; it also escapes from a simple incision. In some lungs there were small groups of grey granulations, not mixed with red, and surrounded by healthy tissue. This was the general description of changes in pneumonia, but there were some peculiarities worth noticing. The diseased lung, especially if it belonged to the first type, (see No. 1,) generally was considerably increased in size. The disease, whether consisting of congestion, hepatization, or suppuration, occupied generally a very large portion of the lung, so that it was extraordinary how life could have been carried on. In one body, the right lung and inferior lobe of the left were throughout in a state of grey hepatization, whilst the upper lobe of the left lung was gorged with blood and frothy bronchial secretion. The granulations were often in groups towards the posterior border of the lung.

The deposition of semi-concrete matter without granulations was never observed except in lungs of the third type, and in these red or grey granulations were never observed. They only existed in lungs of the first and second type, and in these they varied, being regular and distinctly circumscribed in the first, which is characterized by the regularity and rounded form of the cells, and irregular and almost confluent in the second, where the cells are of a more irregular figure.

From these facts it may be inferred, 1st, that the pus, which is capable of being displaced by the pressure of the nail, is seated externally to the air-cells; otherwise, instead of its passing into the interior of the parenchyma, it would escape in isolated drops from the incised surfaces. It is in a situation analogous to that of the air in intervesicular emphysema of the lungs. The sanguineous congestion which attends it (as desiccation proves,) consists of engorgement of the vessels ramifying between the cells. 2d. The granulations, on the contrary, appear to be seated in the cells themselves, as they are rounded or irregular according to the peculiar change in the anatomical structure of the cells, and are not observed in the third type, where the cells are completely disorganized. Compression and desiccation give further proofs of this fact; for by these means the granulations cannot be changed, and the cells cannot be made to reappear, except in those cases where the grey granulations are not friable; where, when compression is employed, small drops of pus escape from the nearest bronchial tubes, and the granulation disappears; thus again proving its situation. The impossibility of removing the red granulations and those grey granules which are friable, by means of compression, &c., shows that they are not merely produced by cells distended with fluid, but that they are solid.—The next enquiry is as to the nature of these changes.

1. Hepatization without granulations.—M. Piorry has demonstrated that, owing to the diminution of the vital contractility of an organ, and in proportion to its spongy parenchyma, the blood abandoned partly to the laws of gravity may, notwithstanding the motions of circulation, accumulate in dependent parts, and stagnate there. This can be constantly verified at the Salpetrière. It renders it difficult to judge whether congestion is active or passive. Friability is not a proof that the congestion is active; for, although it is an effect of inflammation, yet it is sufficient that a patient, or even a corpse, should lie some time on one side to produce both congestion and friability of the corresponding part of the lung. Even among old asthmatics, who pass the last days of their life in a sitting posture, the base of the lungs is very commonly congested and friable. The idea of inflammation must not, on the other hand, be rejected from the situation of the lesion, as granular pneumonia undoubtedly occurs in some instances at the posterior edge of the lungs. The congestion may be determined to be of an inflammatory nature when it occupies the anterior surface on the whole extent of the organ, particularly if no obstacle to the circulation exists in the heart or great vessels. But, under such circumstances, MM. H. and D. have only seen the first degree of congestion, and never impermeability without granulations. The nature of this latter change must then be judged by the symptoms; and, in many cases, their sudden attack and acuteness left no doubt of the inflammatory nature of the lesion. When suppuration and friability are united, there can be no doubt that they are the effect of inflammation. The deposition of semi-concrete matter is also an inflammatory change.

2. Impermeability with granulations.—Both the red and grey granulations are undoubtedly the result of inflammation, as is proved by their situation and mode of formation.

From the preceding remarks it appears established that, among the old, there are two kinds of pneumonia. The first, marked by congestion and impermeability without granulations, followed by the secretion of pus in the interlobular spaces, is seated externally to the bronchial canals in the laminated tissue separating them. The second kind occupies the canals themselves, and is owing either to a granular engorgement of their sides, or to a deposition of pus in their cavities. If the cavities are very irregular, granulations cannot be formed. The first kind of pneumonia may be called intervesicular, and the second vesicular.

*Frequency of Pneumonia in the Old.* The difficulty of distinguishing the disease renders rigorously exact statistics impossible, but the following calculation shows that it is the most frequent and dangerous of the acute diseases of the aged. Of 636 cases of all kinds occurring in the Hospital of the Aged during the periods of these investigations, there were 110 dissections in which there was merely congestion of the base of the lungs produced after death, the patients having had no symptoms of pneumonia, but dying of other diseases; 370 patients were cured after having had symptoms of congestion, recognized both by auscultation and percussion; but, it is true, at the dependent part of the chest, and almost always after prolonged decubitus, so that the inflammatory nature of the congestion might sometimes be contested: fifty-three patients recovered from pneumonia attended by such marked symptoms that it was impossible to doubt its nature; fifteen patients had well-marked symptoms, but no examination could be made: in eighty-eight fatal cases, dissection displayed sanguineous congestion of the lungs reaching the degree of complete impermeability or of suppuration.

Of these eighty-eight cases, there were eighteen only of non-granulated or intervesicular pneumonia, and purulent infiltration in five. The remaining seventy had well-marked granulations or vesicular pneumonia; so that the vesicular was in proportion to the intervesicular pneumonia as four to one. With regard to situation, if those cases are omitted where it was not observed, and where the base of the organ only or the whole lobes were invaded, it appears that the non-granulated engorgement (intervesicular,) occupied constantly the posterior border, whether with or without suppuration, and that the granulated form (vesicular pneumonia,) occupied the anterior border twelve times, and the posterior twenty-five times. Of these twenty-five cases there were fifteen of pneumonia of the inferior lobe, eight of double pneumonia, and only two confined to the upper lobes; whilst all of the twelve cases were of the superior lobes, except two divided between double pneumonia, and pneumonia of the lower lobe. This will be considered fully under the head etiology.

*State of the Bronchi.* In all cases of pneumonia of the old, the bronchial tubes are red from the epiglottis to their minute ramifications, the tint augmenting as they approach their terminations. There are but few old women whose bronchial membrane is not reddened, whatever may be the kind of death. The quantity, often enormous, of mucus which fills the trachea and even obstructs the larynx is worthy of notice. It is thick, viscid, opaque, of a dull white or grey, sometimes yellow or greenish, and occasionally bloody. This latter colour was chiefly remarked in the months of April of the two years during which the investigations were conducted.

*State of the Pleura.* Of sixty cases of vesicular pneumonia, there were marks of pleurisy in thirty-eight. Of these thirty-eight, there were eighteen in which the left pleura was inflamed, coinciding sixteen times with pneumonia of the same side, and twice with double pneumonia; fourteen times the pleurisy was in the right side, in thirteen of which there was pneumonia of the right side, and in one on the left. Six times there was double pleurisy; of these there was double pneumonia in five cases, and pneumonia of the right side only in one.

*Archives Génerales de Médecine. Mars,* 1836.

---

*On Gangrene of the Lungs in the Insane.* By J. Guislain, M.D., Senior Physician to the Hospital for the Insane at Gand.

M. Guislain's attention was called to this disease by observing that the breath of a patient, who obstinately refused his food, smelled exactly like the cavity of the chest of a former patient whose lungs were gangrenous; and, after death, the same lesion was discovered. He thus connected together the obstinate refusal to take food, fetor of the breath, and gangrene of the lung; and subsequent experience proved that the two former were symptoms of the latter. Fetor of the breath is also the consequence of prolonged abstinence, and of pulmonary suppurations, but the smell from gangrene of the lung is altogether peculiar. The obstinate refusal to eat, M. G. considers as an occasional cause of gangrene; it occurs in one-ninth of the insane, and, in more than a thirtieth part of these patients, no care or management will conquer the repugnance. They live twenty, thirty, fifty, sixty days without any food, drinking only cold water; some fast the first days of the week and eat on the others. In but few instances does this arise from a notion that the food is poisoned; it is generally owing to some caprice of the will, dependent on a painful impression. Various fancies confirm this; a child when sulky, and a woman when jealous or spiteful, will not eat: even animals, after losing their master or companion, occasionally refuse all food, and starve. To the debility arising from impoverished blood M. G. attributes the pulmonary disorganization, and he illustrates the influence of a supply of poor blood on the lungs by the effects of the rigorous fasting which some religious communities undergo, the defective and insufficient food of prisons and some charitable institutions, and prolonged abstinence after acute diseases, in producing numerous chronic pulmonary complaints, which (if curable) will only yield to nutritious food and tonics.

The following case illustrates the progress and symptoms of this disease.

Case. In the late political disturbances, an intelligent woman, æt. fifty-four, leading a retired life, was much affected by seeing some armed men fighting below her window. The shock was followed by mental alienation, and she refused to take food. During nine days no persuasions of her family, who put before her food of all kinds, could induce her to eat. From the alteration in her features, her emaciation and melancholy, her family were alarmed, and she was placed in the Institution, the 4th February, 1831, a month after the disease commenced. During this time she had only taken a little milk-soup and weak broth. By the colour of her face only, M. G. recognized her refusal to take food. It was of a brick-red; the cheeks, end of the nose, and lobules of the ears were of a deep brown; pupil dilated; sclerotica of a brilliant whiteness, approaching blue; hair, previously unctuous, was dry, and its colour deteriorated, as well as that of the iris. By force alone, a cup of milk or broth was occasionally taken; but she passed from a state of melancholia to mania; the emaciation frightfully increased, and the face became brown, and the lips, hands, and feet livid as in cyanosis. The smell of the breath was unbearable; expectoration brown, reddish, and streaked with clear blood in considerable quantity, but without pus. The face was so changed that she appeared like a decrepit old woman. She gradually sunk, but during the last few days took the food which was given to her.

*Examination of the Body.* Brain and membranes, and abdominal viscera, healthy: gall-bladder filled with black bile, and the spleen and mesenteric vessels full of very dark blood. In taking out the left lung, M. G.'s fingers penetrated its substance, and there was an insupportable smell. Its posterior surface, towards the upper lobe, was very black, with green and brownish spots: the tissue beneath was so degenerated as to be broken down with the least force: it was infiltrated with a black fetid cruor, like that exhaled from a mortified limb, with here and there purulent flocci. A spherical mass of five inches diameter was reduced to this putrid condition. The bronchi were filled with a reddish, frothy, fetid fluid. Right lung not affected.

M. G. has examined thirteen patients who died of inanition, and in nine of these there was gangrene of the lungs. In one case both lungs were affected; the left lung in seven; and the right in two. Once the gangrene was confined to the anterior surface, but in all the other cases to the posterior and upper part, nearer the spine than the lateral region of the thorax. The pulmonary tissue around the gangrene was injected, but it appeared to be rather the effect of the irritation than the cause of the mortification. In no case was there pain, cough, or dyspnœa; the temperature of the skin was rather cool than hot, and the pulse slower. In all the patients who abstained from food for any length of time, the peculiar hue of the skin was observed which was described in the above case, together with the appearance of premature old age. In no case was the stomach found to be inflamed; it presented no morbid appearance.

Physiologists all agree in stating that, in ordinary cases of starvation, the stomach is inflamed, as if reaction took place: but, among the insane who voluntarily starve themselves, there is no such reaction, no pain, no cardialgia, and, as they affirm when convalescent, no sensation of hunger. There is none of that debility which comes on invariably in the other cases. Thus, maniacs enter the hospitals after having fasted twenty, thirty, forty days; they walk about, and exercise themselves in different ways, and, although extremely emaciated, live for months, or even years, only swallowing from time to time some mouthfuls of broth. Not only is there no sensation of hunger, but food is either not at all or very imperfectly digested. One patient who had thus refused food, took some at eleven o'clock, and committed suicide at seven; the contents of the stomach were found to be unchanged. The absence of prominent symptoms in this gangrene of the lungs is owing probably to the same want of sensibility as is seen in the digestive organs. The lungs do not transmit to the brain the expression of their sufferings; and there is none of that oppression in breathing, violent cough, and dangerous general symptoms which are observed in ordinary individuals suffering from the same local disorganization. The torpor of the par vagum will explain the absence of symptoms both when the lungs and stomach are affected. It also explains the enormous doses of medicine which are tolerated by the insane, and the obstinate torpidity of their bowels. The nerves of sense are equally torpid. They bear without inconvenience the extremes of heat and cold, and the actual cautery hardly is felt: loud sounds in their ears do not disturb them, and they can look at the sun without blinking.

In this inert state of the stomach, very light food is alone suitable. Neither wine nor soup agrees; but milk, either by itself or with the yolk of eggs, is very useful: on this food M. G. has preserved life for two years. Sometimes whey or barley water should be given at first. The patient's obstinacy more than once has given way after taking a few spoonfuls of milk or broth.

It is an important practical fact that the privation of food has a bad influence on the minds of the insane; they become more and more taciturn, and melancholia often passes into mania. In general a restorative diet produces an amendment.

Too much conciliation is injurious. Persuasion is useless and loses time, and energetic measures are necessary at the least opposition. Very frequently, in a few days, such measures completely remove the disgust for food. The following cases are valuable as regards prognosis, corroborating the opinion of Laennec, that gangrene of the lungs is not always beyond the power of medical skill.

Case. Marie de L., æt. twenty-eight, whose father, uncle, and two brothers were insane, was in a state of confirmed melancholia. After two months she refused all food: after three days' abstinence, force was employed, and, by means of a tube passed down the œsophagus, a very little liquid food was given. The peculiar colour of the face appeared; the strength declined, and, after two months of complete abstinence, she had fetid expectoration of a reddish and thin brown colour, without previous cough or dyspnœa. She then spontaneously began to eat, and gradually recovered her health and sanity. Two years afterwards she was readmitted; she refused to eat and had symptoms of pulmonary disease, of which she died. There was gangrene of the left lung.

Case. A young man affected with melancholia refused all nourishment: if force was used he swallowed the food, but immediately excited vomiting by thrusting his fingers into his pharynx; and, when that was prevented, he managed to vomit by contracting the abdominal muscles. He continued these practices for many months, and gradually sunk. Face of a brown colour, lips livid, breath smelled unbearably; a pint daily of reddish expectoration. When almost at the point of death, he suddenly determined to take some milk and broth after a threat to burn his pole with a red-hot iron. Gradually but very slowly he improved, and eventually was completely restored to bodily and mental health.

*Gazette Médicale de Paris,* 16 *Janvier,* 1836.

---

*On the Nature and Mode of Action of Cantharides.* By Dr. Domenico Nardo, of Venice, and Dr. Tommaso Pullino, of Alba.

[We regard as most valuable every fact that tends to illustrate and establish the physiological and therapeutical action of medicines on the animal system; and we are rather disposed to direct attention towards the more extended and closer investigation of the properties of our old remedies, than to hunt after new ones of doubtful efficacy. We have yet much to learn respecting our best known medicines, and certainly respecting that which is the subject of the following pages which contain the more essential parts of two long memoirs.]

*First Memoir*. By Dr. NARDO.

1. Cantharides, when chewed, have not that acrid taste which has been assigned to them, solely from analogy.

2. They will act in an entire state or in powder.

3. The green portion is the only active part of the fly.

4. In this green portion alone is found the *cantharidin*.

5. There is no other poisonous principle existing in the fly.

6. The green principle is analogous to that found in the involucra of other insects having no vesicating powers.

7. The other principles, as the black and yellow substances, are produced by other insects, and are modified by the process followed in their extraction.

8. Cantharidin is a neutral body, unchanged by acids or alkalies; soluble in cold æther, creosote, oils and fats, and in boiling nitric acid and alcohol.

9. It has neither smell nor taste; but, if a small quantity be laid upon the tongue and pressed against the palate, it produces after a time a scalding sensation. The same thing occurs if it be dissolved in æther or oil, which, by aiding its absorption, increases its powers.

10. A very minute quantity of cantharidin is sufficient to excite vesication; for its farther action is arrested the moment that the elevation of the epidermis by the serum removes the absorbents from their contact with it. The vesication, therefore, is not increased either by a larger quantity or longer application of the cantharidin.

11. Cantharidin does not vesicate by irritating or producing any sensible inflammation; (?) its action upon the cutaneous system appears to be limited to the lymphatics, or to a slight stimulus confined to the cutaneous layer below the epidermis, so that the nervous and sanguiferous systems do not suffer at all during the process of vesication. (?)

12, 13, 14. Cantharidin applied to the denuded cutis induces a serous exudation, followed by an atonic suppuration; and acts in the same way that it does on the outer surface, its action is most speedy and painful upon parts most supplied with sebaceous and mucous follicles.

15, 16. Cantharidin is not decomposed when absorbed, and its absorption ceases as the vascular action of the cutis is excited.

17-20. Being carried through the system in an undecomposed state, the cantharidin is eliminated like other foreign matter; but, if the quantity be great, it accumulates in certain parts, and produces its effects according to the nature and susceptibility of the organ. Its stimulating effects upon the urinary passages, and its boasted aphrodisiac powers, are not peculiar properties, but result from the effects of its primary action. Thus, carried along with the urine, it attacks the prostate follicles, producing a state approaching to vesication; and the urine, by increasing this irritation, produces the symptoms of priapism, ischuria, &c. Its poisonous action upon the alimentary canal is produced in an analogous manner, but gives rise to more extensive sympathies.

21. Camphor has no power as an antidote to cantharides.

[Dr. Nardo has given cantharides in various kinds of dropsy, but could never perceive any effect from them. The effects as an aphrodisiac are very uncertain, and usually are only exhibited when they are taken in dangerous doses. The author rather prefers in paralysis of the bladder and incontinence of urine, a weak injection of cantharides.]

*Antologia Medica*, No. VI. Giugno, 1834.

*Second Memoir*. By Dr. PULLINO.

1. Two grains of cantharidin, given at once to a middle-sized rabbit, produced paralysis, coldness, and death in three hours.

2. A grain and a half dissolved in milk caused the same symptoms, and death in an hour and a half.

3. The same dose with m. xv. of cherry-laurel water caused instant death. The rabbit had five hours previously taken m. xx. without injury. Heart empty and flaccid, stomach pale.

4. A fourth rabbit took two grains in solution, with the same symptoms and convulsions of the hind legs. It then took a few drops of ammoniated æther, and one grain of acetate of morphine, at two doses. It revived, but was not lively, and died in twelve days. Stomach reddish here and there; meninges injected.

5. Two dogs of the same age and size, took, the one ten grains of cantharides in decoction; the other twelve grains in powder. The former was paralysed, and died; and, on examination, presented no inflammation of the stomach. The other tried to vomit, was distressed, writhed, and moaned. He was killed in six hours, when the poison was found undigested, and the stomach reddish.

6. Three more rabbits were killed with cantharidin, and two with cantharides. The stomachs of those that drank after taking the poison were uninjured, and the inflammation in those that did not drink was too slight to account for death.

7. The author took two grains of cantharidin at two doses, fasting, and felt a universal shivering and chill down the spine, skin pale, head oppressed, and in one minute the pulse fell five beats. Urine copious in a quarter of an hour afterwards.

8. A fortnight subsequently, he took two grains at four doses. After the second dose he felt a dull pain in the head, and at the next a little vertigo, the skin being cold and clammy. The pulse, after violent action, lost seven beats in a minute. Urine scalding and copious, although but little fluid had been drunk. In the afternoon he took some alcohol, and then ten drops of ammonia in water, when the vertigo ceased and the urine by midnight no longer scalded. Unusual weakness next day.

9. The following are notes of some cases of disease in which cantharides were exhibited:

(1.) *Pleuropneumony.* After two bleedings, the pain continued with bloody sputa of unhealthy consistence. Three grains of cantharides in solution, gradually increased to ten, were taken daily. Continued sweats, urine not increased; sputa healthy, pain gone in sixteen days; eighty-five grains taken.

(2.) *Carditis.* 112 grains taken in twenty days; urine scalding at first, very copious and turbid afterwards.

(3.) *Beating of arteries* of left side of head preventing sleep; had been treated by many bleedings. The patient took one grain of cantharidin in four doses at short intervals. Vomiting, pulse small, rapid, rigors, vertigo, torpor of lower extremities, and other signs of depression. Æther, opium, wine and generous diet, produced a cure.

(4.) *Intermittent Fever* threatening suffocation. Bleeding during the paroxysm, and quinine, were followed by ardent and continued fever, dryness of skin, pains in loins, and ischuria. Fifteen grains of cantharidin taken in eight days at first increased the pain, but not the fever. At first, urine bloody, afterwards *bluish,* then turbid and abundant. Sweat came on soon afterwards, and a cure followed.

(5.) *Lumbago.* Cantharides rendered the pulse hard. The patient had had gastritis just before. Cantharides applied to the epigastrium denuded of the cuticle did not lower the pulse. Bloody urine, but no improvement.

(6.) *Phlegmasia dolens* in a girl with amenorrhœa. Cantharides cured the swelling, but did not restore the uterine functions.

(7.) *Puerperal metroperitonitis* with lumbar pains and dysuria, cured by cantharidin, baths, and mercurial frictions. Urine copious, with some clots of blood.

(8.) *Anasarca* with congestion of the spleen, of three years standing, cured by mercurial frictions and cantharidin, carried to six grains per diem. Urine copious, much viscid perspiration, very slow pulse. The patient fell into a very weak state, which was removed by the use of æther and sherry.

*Annali Universali di Medicina. Settembre,* 1835.

---

*On the Softening of the Mucous Membrane of the Intestinal Canal in Children.*
By Dr. Droste, of Osnaburg.

Dr. Droste coincides in opinion with Andral and others that *ramollissement*, or softening, is an idiopathic disease which may primarily affect all the tissues of the body. The mucous membrane of the intestinal canal of children is peculiarly liable to undergo this process at the commencement of the periods of weaning and teething. Romberg, out of fifty cases of this affection, found that

| | | | | | | | |
|---|---|---|---|---|---|---|---|
| 6 | occurred | from the | 1st | to the | 3d month | (inclusive). | |
| 17 | — | — | 4th | — | 6th | — | — |
| 7 | — | — | 7th | — | 11th | — | — |
| 14 | — | — | 1st year to the 2d year. | | | | |
| 6 | — | — | 2d | — | — | 5th — | |

According to Dr. D., the first symptoms of the disease are frequent stools of a greenish, watery, mucous matter, mixed with yellow flakes, and of an acid nature. It is further accompanied by vomiting of acid matter, cough, impeded respiration, anxiety, perpetual restlessness, frequent crying out and moaning, coldness of the face and extremities, emaciation and debility, and, at last, by convulsions and comatose symptoms. Fever may be present at the commencement, but never lasts till the termination of the disease. The epigastric region is sometimes a little inflated, but never tense; it may be pressed without pain, and the skin is pale and relaxed.

The softening process, of which the above are the symptoms, may be divided into three stages. In the first, the mucous membrane preserves its texture, but loses its normal consistence. This may be the case with the whole of the membrane, or only with patches of it. In the second stage, it is converted into a thin, soft, gelatinous, nearly transparent, matter, which can be wiped away with a sponge, or dissolved by water poured upon it. In this stage, continuity is maintained by the subjacent membranes, though it will always be remarked that these, too, are softer than in a healthy subject. In the third stage, no trace of organization is left in any of the membranes; the intestines are perforated in different places to a greater or less extent, and the internal surface, which is sometimes of a dark red colour, presents here and there traces of extravasated blood.

Most of the modern German pathologists are of opinion, that the cause of this disease is an affection of the nerves which supply the coats of the intestinal canal. This position is principally founded on the celebrated experiments of Camerer,* in which, after cutting, on both sides, the sympathetic and par vagum of a rabbit, he found the coats of the intestinal canal softened in precisely the same way as in the diseased human subject. Basing his views on this fact, Camerer concludes, that the cause of the disease under consideration is an inflammation of the above-mentioned nerves, terminating in paralysis. But Droste and the later writers, whilst they allow that the nerves are primarily affected, deny that they are inflamed. They content themselves with asserting that they are in an anormal condition, which may be either of excitement or depression. They ask—and in vain—for the anatomical signs of inflammation. Moreover, the remedy for this disease, the efficacy of which they describe as remarkable, and as acknowledged throughout Germany, proves alone that the nature of the latter cannot be inflammatory. This remedy is the *muriate of iron*. Pommer relates two cases of its decided success. One of his patients was a child of six months old, to whom he gave two scruples of muriate of iron within a week, in a decoction of marshmallow. The other, a child of four weeks old took twenty-four grains in eight days. Both were allowed scarcely any nourishment, except a few tablespoonfuls of warm milk or gruel. Hergt gives, in these cases, musk in conjunction with iron. The day after the first exhibition of these remedies, sickness and diarrhœa had both disappeared. He prescribes also liniments for the epigastric region, and warm aromatic fomentations for the whole abdomen.—*Zeitschrift für die gesammte Medicin, &c. Hamburg.* 1 Band, Heft 4. 1836.

* See "Versuche über die Natur der krankhaften Magenerweichung, von Camerer. Stuttgart, 1828.

*On the Treatment of Tympanites with Camphor, Musk, and Ammoniacum.*
By Drs. TRADINI and SANTOLI.

1. FROM the inefficacy of other means in this obstinate complaint, Dr. Tradini was induced to try camphor in considerable doses.

A man, of forty-five, was annoyed for a week previously, from his abdomen being tumid, tense, and resonant; an intercurrent affection on an old obstruction of the liver. The following pills were prescribed:

℞. Camphoræ subtiliter pulver. gr. vj.
Extracti cinchonæ offic. gr. viij. M.
fiat pilula omni quarta horâ sumenda.

He was ordered a nutritive diet, and frictions with warm flannel over the abdomen. Before he had taken twenty pills he was entirely relieved, but he was directed to take one daily for the following eight days. During the use of the medicine, he had a sensation of great heat in the intestines, with obstinate constipation.

The second case was that of the famous improvisatore, Pistrucci. The abdominal distention had lasted a month, and no remedies had succeeded.

℞. Camphoræ rasæ pulver. gr. vij.
Ext. gummosi Cinchon. off. gr. viij. M.
fiant pilulæ duæ. Sumantur omni quartâ horâ die et nocte per dies tres.—Animal diet, and twelve ounces of port wine.

At first the pills produced great heat; on the third day the distention had disappeared. Two pills were continued daily for the next four days, when the strength and appetite were re-established.

The last case was that of a boy, nine years old: the tympanites had lasted a month, with considerable emaciation and debility. He was covered with warm flannel, and ordered to take nutritive broths, barley water, and the following pills:

℞. Camphoræ rasæ subtiliter pulv. gr. ij.
Squillæ maritimæ pulv. gr. ½.
Ext. G. Cinchonæ offic. gr. iv. M.
fiat pilula; dentur tales n. 6, sumat unam omni tertia horâ ante nutrimentum.

After taking six pills, the patient made much urine. After three days, the improvement was marked; and three other pills, continued daily for a short period, procured the most gratifying establishment of the health, increasing the appetite and restoring the strength.

II. In consequence of the suggestion of Dr. Tradini, another medicine, having probably somewhat of the same action as camphor, has been recommended by Dr. G. Santoli, of Naples, who has witnessed repeated instances of its efficacy. The prescription was given him in 1812, by an old practitioner, who had inherited it. Three grains of musk, and twelve of gum ammoniac, are made into three pills by means of some soft extract, and the patient is to take one every morning, noon, and night. In 1813 he prescribed it, with immediate benefit, to a lady who was much troubled, at the time of the final cessation of the menses, with a highly tympanitic state of the abdomen, which had resisted all treatment. Two years afterwards he prescribed it with equal success to a countryman, æt. 65, who was prevented by tympanitis from working, and was consequently in great poverty. This treatment was in unison with the Brunonian theory, then in fashion; but, from Dr. Santoli having afterwards adopted Brouissais' doctrines, he gave up his musk, notwithstanding his previous experience of its efficacy, until 1831, when he was consulted by a woman, æt. 35, who for four years (after having had many attacks of intermittent fever,) had suffered from a disease for which she could get no relief. She was extremely emaciated; and the abdomen only was swollen, strongly distended, and very hard. The sclerotica, as well as the whole body, was yellow. The medical men whom she had previously consulted had told her that the liver was the organ diseased, and it appeared from her prescriptions that antiphlogistic and lowering remedies had been exhausted in vain. Dr. Santoli now thought that it was better to trust to his former experience than to the commonly adopted theory, and prescribed consequently the pills of musk and ammoniacum. In ten days she was much improved; the abdomen was soft, and the colour and functions more natural. On the fortieth day she was perfectly restored to health. The medicine acted as an aperient and sudorific.

*Il Filiatre Sebezio.* Vol. ix. 1835.

---

*An Enquiry into some Points of the History of Leucorrhœa.*
By Dr. MARC L'ESPINE.

THE statistical facts which form the basis of the present essay were collected in the Venereal Hospital of Paris. The author insists on the necessity of employing the speculum, as the only means of acquiring a correct knowledge of the disease. In several females, who stated that they never had any leucorrhœa, he found a discharge from the os uteri, on employing the speculum. The cases which he has collected of uterine discharge are the most important, and the connexion which he has found to exist between the matters secreted and the condition of the os uteri are worthy of a short analysis. Of 193 cases of uterine discharge which were examined with a speculum, there were twenty-three in which the os uteri was dry, and the vagina was equally free from any matter which could be traced to a uterine origin. In forty cases, there was only a single drop pendent at the os uteri. In the remaining 130, the discharge was more abundant. This discharge is not necessarily stopped during pregnancy. In a large proportion of cases in which the menses were somewhat retarded, the discharge was also found wanting. The fluid is either watery or more or less viscid, and in the latter case of various characters; sometimes transparent, at others opaline, streaked, opaque, white, or yellow. The condition of the cavity of the uterus is necessarily unknown; but its orifice may be either healthy or surrounded by a rosy circle; this circle may be of a deep red colour or bloody, or the redness may be granular without erosion; or, lastly, the circle may be eroded and ulcerated, with a smooth or granulated surface. The fact of all these cases having been assembled in the Venereal Hospital renders it doubtful how far these discharges may be dependent on a syphilitic cause, or originate in simple chronic inflammation. But, as illustrating the connexion between the condition of the parts and that of the discharge, the facts are still of some interest. From a numerical comparison, the author found that the watery and transparent albuminous discharges existed in the majority of cases in which the orifice was healthy, in one half of those where it was surrounded by simple redness, and only in a fourth of the cases in which there was vivid redness or ulceration. The opposite may be said of those discharges which were striated, semitransparent, and opaque. However, the nature of the discharge is not solely dependent on the condition of the orifice, nor does a similar condition always produce a similar discharge; for an ulceration of the cervix, which coincides most frequently with the opaque, coincides occasionally with the aqueous or albuminous discharges, and those which are opaque and striated are sometimes met with when the cervix is perfectly healthy. It is probable that the aqueous and albuminous discharges belong properly to leucorrhœa; whilst the addition of streaks of purulent or opaque white fluid, depends rather on an inflammation which is either simple or syphilitic. So little that is important can be concluded from the author's examination of the vagina and its discharges, that it is scarcely worthy of notice.

*Archives générales de Médecine,* tome x. *Fevrier,* 1836.

---

*On the Use of Chloride of Soda in Intermittent Fevers.* By Dr. GOUZEE,
First Physician of the Military Hospital at Anvers.

DR. GOUZEE was induced to try this medicine, first recommended by Dr. Lalesque, as its cheapness would render it (if efficacious) very valuable to the poor inhabitants of marshy districts, as a substitute for quinine. The dose prescribed was half a drachm of chloride of soda in four ounces of distilled water, to be taken by spoonfuls between the fits, and so that the last doses should be swallowed shortly before the next paroxysm was expected. The patients were restricted to a light diet, and confined to their beds, or at least their chambers. Ten cases are reported of ague: in two the intermittent yielded immediately; two others were cured after a slight return; in one there were four attacks, gradually diminishing; in two cases the severity of the paroxysms abated, but it was thought necessary to have recourse to sulphate of quinine; in two others no effect was produced, and in one the disease was aggravated. Dr. G. thinks that these cases prove the febrifuge properties of the chloride of soda to be less marked than those of sulphate of quinine, and therefore that it should not be trusted to except in the slighter cases, and where the patients are readily susceptible of the effects of medicine, as women and children. *Revue Médicale. Fevrier,* 1836.

---

*On the External Application of Croton Oil in Affections of the Larynx.*
By Dr. Romberg.

The following cases prove the peculiar efficacy of this species of counter-irritation in affections of the organs of voice; a fact observed by many.

Case i. A fisherman, æt. 34, lost his voice after exerting himself greatly in saving some individuals from drowning. There was no reason to suspect any disorganization of the larynx. Blisters, vapour baths, &c. were tried without effect. Frictions of croton oil were directed over the larynx, to be repeated as soon as the eruption declined. On the twenty-first day of this treatment he began to recover his voice, and regained it completely.

Case ii. A girl, æt. 18, suffered during seven weeks with hoarseness, succeeded by aphonia, the consequence of a sudden chill. Leeches, emetics, and irritating frictions produced no relief; but, after the third application of croton oil, an eruption appeared, and she immediately regained her voice.

Case iii. A woman, æt. 38, complained for twelve months of a sensation of pressure in the pharynx, as if the neck were squeezed, rendering deglutition difficult: there were no other symptoms. Many remedies were tried without benefit. Three drops of croton oil were rubbed in, and, after the third application, an eruption appeared on the neck, nucha, chest, and face, which was followed by erysipelas. The patient entirely recovered.

Dr. Romberg never found that the external application of croton oil had a purgative effect, but he never applied it to the abdominal integuments.

Dr. Otto reports, in the same journal, the case of a woman affected with sciatica, for which frictions with croton oil were made on the thigh, and the whole body became red and covered with vesicles. Dr. Otto never observed its purgative effect when thus applied.

*Wochenschrift für die gesammte Heilkunde.* 1835.

---

*Expectoration of Bronchial Polypus, independent of Croup.*
By Professor Casper.

Dr. Cheyne has described two kinds of bronchial polypus, unconnected with croup; one of which appears to be but a coagulum of blood, and is associated with hæmoptysis; the other symptomatic of a chronic disease, of an inflammatory character, affecting a secreting surface: *this inflammation, however, never reaching to the extent observed in croup.* The following case appears to lead to the conclusion, that inflammation and the formation of a false membrane observed in croup are by no means necessarily connected. In this view of the subject alone is the action of many remedies explicable. Dr. Casper justly maintains that the danger of croup is not simply dependent on the mechanical impediment produced by the false membrane, but that it is to be ascribed likewise to a *specific* inflammation, which, in respect to its symptoms and its resistance to the usual remedies, stands in the same relation to common inflammation as do many forms of abdominal inflammation, &c.

A girl, twelve years of age, of a lymphatic scrofulous constitution, was affected, on the 2d of May, by inflammatory catarrh, which yielded to a few leeches and a mixture containing nitre. She left her bed on the fourth day, and expectorated occasionally and without difficulty. On the afternoon of May 7th, a violent cough and suffocative paroxysm unexpectedly occurred, and the patient expectorated a whitish-yellow polypous body, which appeared externally very like concrete fat, was of a firm and tenacious character, was with difficulty torn, and corresponded to the ramifications of the bronchi. During the following twelve days, two and twenty similar substances were expectorated. The first ten were accompanied with violent cough and paroxysms of suffocation; the expectoration of the remaining twelve was very easy, long after the patient had left her bed, when she was quite free from fever, had a good appetite, slept well, and had only a slight hoarseness of voice. This hoarseness had existed many years previously, and still continues.

Two of these bodies were generally expectorated daily; one in the morning, and the other towards midnight. The health of the patient being otherwise good, very little medical treatment was employed.

*Wochenschrift für die gesammte Heilkunde. No.* 1. 1836.

---

*On Incontinence of Urine.* By M. Mondiere.

M. Mondiere has employed the extract of nux vomica in cases of nocturnal incontinence of urine, with very beneficial effects. The case in which its efficacy was most strongly shown is that of a young woman, aged twenty, who, from the age of six years, had constantly voided her urine involuntarily during the night. The use of twelve of the following pills put an end to the incontinence: they were continued until twenty-four grains of the extract had been taken, and, during the year following this treatment, there was no return of the disease. Other successful cases are mentioned.

Extracti nucis vomicæ, gr. viij.
Ferri protoxidi, gr. j. M. fiant pil. xxiv.

*Gazette Médicale. No.* 10. 1836.

---

*Formula for an artificial Chalybeate Water.*

℞. Ferri Sulphatis, ʒss.

Sacchar. albi, ʒiss. Misce, et divide in chart. xij. æq.

D.S. No. 1.

℞. Sodæ Carbonatis, ʒss.

Sacchar. albi, ʒiss. M. et divide in pulv. xij. æq.

D.S. No. 2.

One powder from each of these packets is to be dissolved in a small quantity of water, then mixed and drunk whilst effervescing. Each draught contains about a grain of the carbonate of the protoxide of iron, dissolved in water impregnated with carbonic acid gas, with a little Glauber's salt and carbonate of soda; the carbonate of soda being designedly a little in excess. This is a good substitute for ferruginous mineral waters, where the natural ones cannot be obtained.

*Summarium des Neuesten in der Heilkunde.* 1835.

---

*Cinnabar Fumigations.*

Dr. Venot employs with success the following mode of fumigating venereal ulcers of the throat with cinnabar. After soaking sage-leaves in strong gum-water, the sulphuret of mercury is sprinkled over them, and they are dried in the sun; they are afterwards smoked in a pipe instead of tobacco, and the vapour is thus directly and conveniently applied to the diseased surface. Several cases are reported, to prove its efficacy.

*Journ. de Méd. Pratique de la Soc. Roy. de Bourdeaux. Février,* 1836.

